# Protected characteristics reported in pulmonary rehabilitation: a scoping review

**DOI:** 10.1183/16000617.0236-2023

**Published:** 2024-06-12

**Authors:** Holly Drover, Lucy Gardiner, Sally J. Singh, Rachael A. Evans, Enya Daynes, Mark W. Orme

**Affiliations:** 1 Department of Respiratory Sciences, University of Leicester, Leicester, UK; 2 Centre for Exercise and Rehabilitation Science, National Institute for Health and Care Research (NIHR) Leicester Biomedical Research Centre – Respiratory, University Hospitals of Leicester NHS Trust, Leicester, UK; 3 Both authors contributed equally

## Abstract

**Background:**

An individual's characteristics are reported to influence access, completion and outcomes of pulmonary rehabilitation and may contribute to health inequalities. Many countries have policies to promote equity among individuals’ characteristics, including the UK Equality Act 2010 which lists nine protected characteristics (age, disability, gender reassignment, marriage and civil partnership, pregnancy and maternity, race, religion or belief, sex and sexual orientation).

**Objectives:**

To describe the extent to which UK Equality Act 2010 protected characteristics have been collected and reported in UK studies and audits of pulmonary rehabilitation.

**Methods:**

A scoping review following the Preferred Reporting Items for Systematic Reviews and Meta-Analyses for Scoping Reviews guidelines was conducted using five databases. UK studies and audits collecting data on pulmonary rehabilitation from 1 October 2010 (date of Equality Act 2010 inception) were eligible. The protected characteristics collected and how they were reported were extracted.

**Results:**

Out of 45 included studies and audits (41 studies and four audits), 98% (k=44) reported age. Sex was reported in 40% (k=18), and 20% (k=9) reported gender with only male and female categories. Half (50%, k=2) of audits reported gender with male, female and transgender categories. Race was reported through ethnicity in 2% (k=1) of studies and 75% (k=3) of audits. No studies or audits explicitly reported disability, but all reported measures indicating disease severity (*e.g.* forced expiratory volume in 1 s % predicted: 67%, k=30). No studies or audits reported marriage and civil partnership, pregnancy and maternity, religion or belief or sexual orientation.

**Conclusions:**

Protected characteristics are not commonly reported or are inconsistently reported in UK pulmonary rehabilitation studies and audits. Without reporting these characteristics, health inequalities in pulmonary rehabilitation will remain unclear.

## Shareable abstract

**Protected characteristics are not commonly reported or are inconsistently reported in UK studies and audits of pulmonary rehabilitation. Without these data, health inequalities relating to pulmonary rehabilitation will remain unclear.** https://bit.ly/3TQO1Hm

## Introduction

Pulmonary rehabilitation is an internationally recommended [1, 2] complex intervention for individuals living with chronic respiratory disease consisting of tailored exercise and education that has demonstrated improvements in health-related quality of life [3] and survival [4]. An individual's characteristics such as age, disability, gender, marital status, religion or belief, sex and race have been found to influence access, completion and outcomes of pulmonary rehabilitation [5–19] and outcomes of chronic respiratory disease [20–22], potentially contributing to health inequalities [2, 23–25]. Health inequalities highlight differences in health between individuals including health status and access, experiences and outcomes of healthcare [23, 26], such as in pulmonary rehabilitation [27]. Systemic factors such as income, housing and geographical location drive health inequalities [28] and often means individuals with certain characteristics will be more vulnerable to its effects. In recent years, reducing health inequalities have become a priority for health services [29, 30] and addressing them is a major challenge for healthcare providers globally [31, 32]. The avoidable cost of health inequalities is estimated to cost European Union member states up to EUR 1.3 trillion every year [33, 34].

Collecting and reporting an individual's characteristics in pulmonary rehabilitation studies and audits consistently allows for the monitoring of potential health inequalities and may provide information on differences in the access, completion and outcomes of pulmonary rehabilitation across individuals [35, 36]. This information can be used to establish whether services are equitable, fair and accessible for all [37, 38] and challenge health inequalities by adapting and improving services [38], including pulmonary rehabilitation [39]. Reporting guidelines such as Consolidated Standards of Reporting Trials, Strengthening the Reporting of Observational Studies in Epidemiology and Consolidated Criteria for Reporting Qualitative Research recommend the reporting of “important” characteristics of study participants; however, no specific characteristics are stated [40–42]. Without consistently collecting the characteristics of those attending pulmonary rehabilitation, effective intervention and service improvements to challenge health inequalities cannot be implemented and the widening health inequalities [28, 43] cannot be addressed in pulmonary rehabilitation.

Many European countries have legislation to reduce inequalities and improve equity and inclusivity [44–51] or are part of intergovernmental organisations (such as the United Nations) that publish antidiscrimination governance [52, 53] on individual characteristics such as age [44, 45, 47–49, 51], disability [44, 45, 47–49, 51], gender identity [48, 49, 51], marital status [46, 47], pregnancy [49], race [44, 46–51, 53], religion or belief [44–51, 53], sex [44, 47, 48, 50, 51, 53] and sexual orientation [44–49, 51]. One example of this legislation is the United Kingdom (UK) Equality Act 2010. This legislation describes protected characteristics of individuals that are illegal to discriminate against [47]. These are age, disability, gender reassignment, marriage and civil partnership, pregnancy and maternity, race, religion or belief, sex and sexual orientation [47].

The UK Equality Act 2010 protected characteristics were used in this scoping review to explore the health inequality related characteristics of individuals collected and reported in pulmonary rehabilitation research studies and audits. Accordingly, the aim of this scoping review was to describe the extent in which UK Equality Act 2010 protected characteristics have been collected and reported in UK research studies and audits of pulmonary rehabilitation to date. This review answers the following questions. 1) To what extent have UK Equality Act 2010 protected characteristics been collected as part of UK pulmonary rehabilitation research studies and audits? 2) How have UK Equality Act 2010 protected characteristics been reported in UK pulmonary rehabilitation research studies audits?

## Methods

### Protocol and registration

The review followed the Preferred Reporting Items for Systematic Reviews and Meta-Analyses scoping review guidelines [54]. The protocol was prospectively registered on Open Science Framework on 15 November 2022 [55].

### Eligibility criteria

The Population, Intervention, Comparison, Outcomes, Study Design and Setting framework was used for article eligibility criteria. Any research study or audit conducted in the UK with adults (aged ≥18 years) participating in pulmonary rehabilitation were eligible for inclusion. The pulmonary rehabilitation programme could be carried out in any outpatient setting (*e.g.* home-based, centre-based, remotely delivered) in the UK [56]. There were no limitations in comparators or outcomes. Both published and unpublished research studies and audits were included; however, case studies and conference abstracts were excluded. Research studies were excluded if they started data collection prior to 1 October 2010 (prior to the inception of the UK Equality Act 2010).

### Search strategy

MEDLINE, Cumulated Index to Nursing and Allied Health Literature, Scopus and APA PsycInfo were searched for relevant articles from 1 October 2010 to 15 November 2022. Searches were updated in January 2024. Preprint server medRxiv and Google Scholar were also searched. The search strategy covered key terms for pulmonary rehabilitation and is detailed in the supplementary material (supplement A).

Search results were exported into Rayyan [57] and duplicates removed. Two independent authors screened the title and abstract of each article before screening the full text for eligibility (H. Drover, L. Gardiner, E. Daynes, M.W. Orme). Any conflicts were resolved through discussion. The reference lists of included articles were checked for possible additional articles.

### Data charting

#### Definitions of protected characteristics

In order to ensure objective data charting, clear definitions of each protected characteristic are required. The UK Equality Act 2010 defines each protected characteristic as follows.

Age: the protected characteristics of an individual's age or range of ages [47].

Disability: the protected characteristic of an individual with a physical or mental impairment that has a substantial and long-term (≥12 months) impact on an individual's ability to complete usual day-to-day activities. Disability also includes if an individual has been diagnosed with a progressive condition and this condition has an impairment on an individual's ability to complete usual day-to-day activities, even if this effect is not substantial [47]. This is likely to include most individuals who are eligible for pulmonary rehabilitation. For the purpose of this review, whether participants or service users had a disability or did not have a disability reported and measures of disability were examined.

Gender reassignment: the protected characteristic of an individual who is planning to undergo, is undergoing or has undergone medical or surgical processes to reassign their sex or an individual who does not wish to undergo medical or surgical processes, but intends to live permanently as a different gender to that which they were assigned at birth [47]. Terminology may include (but is not limited to) trans, transgender and transsexual [58].

Marriage and civil partnership: the protected characteristic of an individual who is married or is in a civil partnership [47].

Pregnancy and maternity: the protected characteristic of an individual who is pregnant [47]. Maternity refers to 26 weeks after giving birth [47].

Race: the protected characteristic of race includes an individual's colour, nationality (including citizenship), national origins and ethnicity [47].

Religion or belief: an individual's religion or philosophical belief, or lack of religion or belief. For a belief to be classed as a protected characteristic it should affect an individual's life choice [47].

Sex: a man or woman as defined by the sex on a legal document such as a passport, birth certificate or gender recognition certificate [47].

Sexual orientation: an individual's sexual orientation towards people of the same sex, opposite sex or either sex [47].

A data-charting form on Microsoft Excel mapped the extracted data and included data items such as year of publication/availability, evidence source (*e.g.* published, preprint), study design (*e.g.* randomised controlled trial, audit), sample size, participant's respiratory conditions and pulmonary rehabilitation delivery method (supplementary material B (research studies) and C (audits)). Whether protected characteristics were reported (yes/no) and how they were reported (*e.g.* categories reported, wording used) were extracted. If not all categories of a protected characteristic were reported (*e.g.* not all age categories for the protected characteristic of age; not both male and female for the protected characteristic of sex; not all participants’ race categories or ethnic groups for the protected characteristic of race); whether the majority or minority category/categories were reported was extracted. Where possible, for the protected characteristic of race, the UK census categories were used to report the data, although it is acknowledged that the categories do not contain all possible ethnic groups. The data-charting form was piloted using five eligible articles and amended before wider use. Data charting was conducted by two authors independently (H. Drover, M.W. Orme), with discrepancies resolved through discussion.

### Reporting results

Results were reported descriptively using k (number of studies) (%). The results for research studies and audits were analysed separately and by each protected characteristic. Figures were created with the data visualisation software Flourish (https://flourish.studio/).

## Results

**FIGURE 1:**
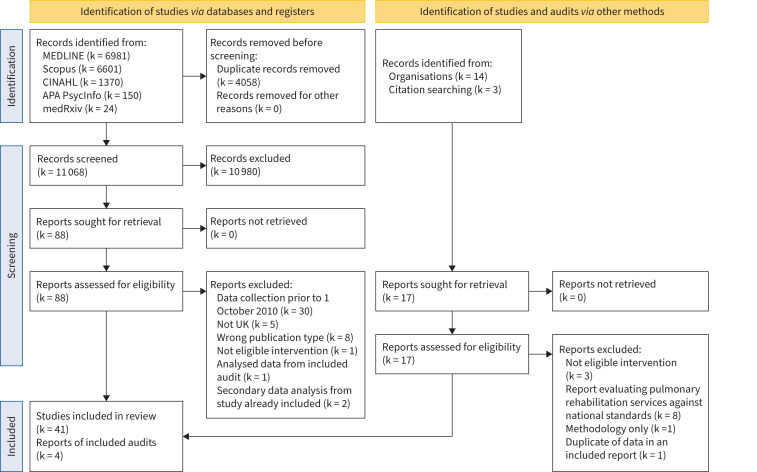
Preferred Reporting Items for Systematic Reviews and Meta-Analyses 2020 flow diagram. Reproduced from [100] with permission. CINAHL: Cumulated Index to Nursing and Allied Health Literature; UK: United Kingdom.

15 126 research studies were identified after the initial database search. Following the removal of 4058 duplicates, titles and abstracts were screened for 11 068 research studies, with 88 meeting criteria for full-text screening, and 41 were included [59–99] (figure 1). Out of 14 eligible National Respiratory Audit Programme (NRAP) audit reports, four audit reports were included in the scoping review [16, 100–102] (figure 1).

### Characteristics of included research studies

Out of 41 included research studies, 35 (85%) included participants with COPD [59–61, 63, 64, 66–69, 71–80, 81, 82, 84, 85, 86–94, 96, 98, 99], five (12%) bronchiectasis [60, 65, 73, 83, 84], five (12%) asthma [62, 70, 72, 73, 84] and three (7%) interstitial lung disease [72, 84, 97] as a primary diagnosis. 38 (93%) research studies delivered pulmonary rehabilitation from a centre (hospital, community or another outpatient setting) [59–71, 73, 76–81, 82, 83, 84, 85, 86–99]; seven (17%) delivered home-based pulmonary rehabilitation [69, 72, 74, 80, 82, 86, 90]; and five (12%) delivered online home-based programmes [72, 74, 80, 82, 86]. The characteristics of included research studies are presented in table 1.

**TABLE 1:** Characteristics of included research studies

First author year [ref.]	Year of data collection	Study design	Mode of pulmonary rehabilitation delivery	Primary respiratory condition	Sample size n
**Aldabayan 2019 [**89**]**	2017–2018	Observational study	Hospital centre-based	COPD	92
**Andrews 2015 [**88**]**	2012–2013	Service evaluation	Hospital centre-based and community	COPD and other chronic respiratory conditions which are not specified	363
**Armstrong, 2021 [**94**]**	2018–2020	RCT	Unspecified centre-based	COPD	48
**Barlow 2020 [**87**]**	2016–2017	Service evaluation	Community	COPD	322
**Bourne 2017 [**86**]**	2015–2016	RCT	Rehabilitation facility or online home-based	COPD	90
**Boutou 2014 [**85**]**	2012–2013	Database analysis	Hospital centre-based	COPD	787
**Bradley 2022 [**98**]**	2020	Qualitative interviews	Community	COPD	9
**Briggs-Price 2022 [**84**]**	2016–2019	Propensity-matched cohort study	Hospital centre-based	COPD, bronchiectasis, asthma, ILD, other restrictive, other	1492
**Chalmers 2019 [**83**]**	2014–2018	Pilot RCT	Hospital centre-based	Bronchiectasis	48
**Chaplin 2022 [**82**]**	2013–2015	Exploratory feasibility study	Hospital centre-based, community or online home-based	COPD	103
**Chaplin 2015 [**81**]**	2011–2012	Retrospective audit	Hospital centre-based	COPD	200
**Chaplin 2017 [**80**]**	2013–2015	Randomised controlled feasibility study	Hospital centre-based, community or online home-based	COPD	103
**Cox 2018 [**90**]**	2015–2016	Parallel-group, pilot 2×2 factorial randomised trial with nested qualitative research and an economic analysis	Hospital centre-based and home-based	COPD	61
**Edwards 2023 [**95**]**	2012–2017	Cohort study	Hospital centre-based	IPF	166
**Finnegan 2023 [**96**]**	2013–2020	Experimental medicine study	Hospital centre-based or community	COPD	72
**France 2021 [**79**]**	2018–2019	Prospective observational study	Hospital centre-based	COPD	112
**Jenkins 2020 [**77**]**	2016–2018	Prospective cohort study	Community	COPD	85
**Jenkins** **2020 [**78**]**	2016–2018	Prospective cohort study	Community	COPD	40
**Jones 2015 [**76**]**	2011–2014	Case–control	Hospital centre-based	COPD	622
**Jones 2014 [**75**]**	2011–2012	Audit	Unspecified	COPD	448
**Jung 2020 [**74**]**	2018	Mixed methods	Online home-based	COPD	10
**Knox 2019 [**73**]**	2017–2018	Service evaluation	Hospital centre-based or community	COPD, bronchiectasis, pulmonary fibrosis, chronic asthma, other	45
**Lewis 2021 [**72**]**	2019–2020	Mixed methods	Online home-based	COPD, ILD, asthma	30
**Maddocks 2016 [**71**]**	2011–2015	Prospective cohort study	Hospital centre-based	COPD	816
**Majd 2020 [**70**]**	2014–2017	Feasibility study of RCT	Hospital centre-based	Severe asthma	61
**McDonnell 2014 [**93**]**	2011–2012	Prospective controlled “before and after” study	Hospital centre-based or community	COPD	52
**Nikoletou 2023 [**97**]**	2014–2015	Randomised controlled pilot feasibility trial	Hospital centre-based	ILD	58
**Nolan 2019 [**69**]**	2012–2015	Propensity-matched cohort study	Community or home-based	COPD	154
**Nolan 2017 [**68**]**	2012–2014	RCT	Hospital centre-based	COPD	152
**Nolan 2022 [**67**]**	2013–2018	Propensity matched real-world study	Hospital centre-based	COPD, IPF	326
**O’Neill 2018 [**66**]**	2014–015	Mixed methods	Hospital centre-based	COPD	49
**Patel 2023 [**99**]**	2018–2019	Cohort study	Unspecified centre-based	COPD	24
**Patel 2021 [**64**]**	2011–2016	Propensity-matched analysis	Hospital centre-based or community	COPD	636
**Patel 2019 [**65**]**	2012–2016	Propensity-matched control study	Hospital centre-based or community	Bronchiectasis	426
**Pavitt 2020 [**63**]**	2015–2018	Randomised parallel-group study	Hospital centre-based	COPD	165
**Ricketts 2022 [**62**]**	2017–2020	Pragmatic RCT	Hospital centre-based	Difficult-to-control asthma	95
**Ward 2021 [**61**]**	2016–2018	Single-arm feasibility trial	Hospital centre-based	COPD	19
**Wynne 2020 [**60**]**	2012–2017	Matched observational cohort study	Hospital centre-based	COPD, bronchiectasis	124
**Yohannes 2021 [**59**]**	2014–2016	Prospective trial	Community	COPD	165
**Yohannes** **2022 [**91**]**	2013–2019	Cohort study	Community	COPD	734
**Yohannes 2022 [**92**]**	2013–2019	Database analysis	Community	COPD	993

RCT: randomised controlled trial; ILD: interstitial lung disease; IPF: idiopathic pulmonary fibrosis.

### Protected characteristics in pulmonary rehabilitation research studies

**FIGURE 2:**
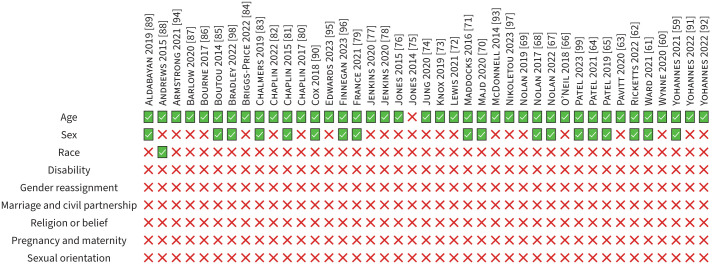
Summary of the protected characteristics reported in the included research studies.

Figure 2 summarises the protected characteristics reported in the research studies. Further reporting details are detailed in the supplementary material (supplement D).

#### Age

Age was the most frequently reported protected characteristic (98%, k=40) [59–74, 76–99]. Age was most commonly reported as mean±sd (80%, k=33) [59–61, 64–74, 76–78, 80­–82, 84–95, 97], followed by median (interquartile range (IQR)) (12%, k=5) [62, 63, 79, 83, 99]. Each of the individual participants’ ages was reported in one (2%) of the research studies [74].

#### Sex

The sex of participants was reported in 44% (k=18) of research studies [59, 61, 62, 64, 65, 67, 68, 70, 71, 79, 81, 83, 85, 89, 90, 96, 98, 99]. Gender was reported in 20% (k=8) of research studies with only two categories (male/female) [66, 72, 74, 82, 87, 88, 91, 94]. In 17% (k=7) of research studies it was unclear if authors reported sex or gender [73, 76–78, 84, 92, 94]; 7% (k=3) used the terms “sex” and “gender” interchangeably [76, 84, 92]; and 10% (k=4) reported either male or female, but did not state if this was sex or gender [73, 77, 78, 95].

In research studies where only one gender or sex category was reported, 64% (k=16) reported the majority [59–61, 67, 68, 70, 71, 77–80, 83, 84, 86, 95, 97]. When a sample was predominantly male, 76% (k=13) reported only the male category [59, 61, 67, 68, 71, 77–80, 84, 86, 95, 97]. When a sample was predominantly female, the female category was reported in 38% (k=3) of research studies [60, 70, 83].

#### Race

No research studies explicitly used the term “race”. Ethnicity was reported in one (2%) study [88]. Ethnicity was reported as n (%) for White British, Irish, White other, Mixed British and African ethnic groups [88]. Three (7%) research studies did not report the ethnicity or race of participants, but discussed participants being predominantly or all “Caucasian” as a study limitation [59, 91, 92].

#### Disability

No research studies explicitly reported that their participants had a disability. 98% of research studies reported measures of disability, including disease severity, exercise capacity or breathlessness [59–97, 99]. Forced expiratory volume in 1 s (FEV_1_) % predicted was the most common marker of disease severity (73%, k=30) [59–71, 76, 77, 79, 80, 82–86, 89–94, 96, 99], incremental shuttle walk test for exercise capacity (66%, k=27) [59–61, 63–71, 73, 76, 77, 80–82, 84, 85, 87, 89, 91–93, 95, 99] and Medical Research Council (MRC) dyspnoea scale for breathlessness (59%, k=24) [60–64, 66–74, 76, 79–82, 84, 85, 95, 96, 99].

#### Gender reassignment, pregnancy and maternity, marriage and civil partnership, religion or belief and sexual orientation

No research studies reported gender reassignment, marriage and civil partnership, pregnancy and maternity, religion or belief or sexual orientation.

### Characteristics of included pulmonary rehabilitation audits

Table 2 presents the characteristics of the included audits. All audits were published by the NRAP, formerly known as the National Asthma and COPD Audit Programme. NRAP reports the data for individuals with COPD who have been referred to pulmonary rehabilitation and that have consented for their data to be entered into NRAP pulmonary rehabilitation audits. Of the four included audits, three (75%) were clinical audits [101–103] and one (25%) was a joint clinical and organisational audit [16]. All audits (k=4) included both centre-based and home-based pulmonary rehabilitation programmes [16, 101–103].

**TABLE 2:** Characteristics of included audits

First author year [ref.]	Year of data collection	Audit type	Mode of pulmonary rehabilitation delivery	Primary respiratory condition	Number of service users
**Steiner 2016 [**103**]**	2015	Clinical	Hospital centre-based, community and home-based	COPD	7413
**Singh 2020 [** 102 **]**	2019	Clinical	Hospital centre-based, community and home-based	COPD	6056
**Singh** **2020 [**101**]**	2019	Clinical	Hospital centre-based, community and home-based	COPD	12 127
**Singh 2020 [**16**]**	2019	Clinical and organisational	Hospital centre-based, community and home-based	COPD	12 127

### Protected characteristics in pulmonary rehabilitation audit reports

**FIGURE 3:**
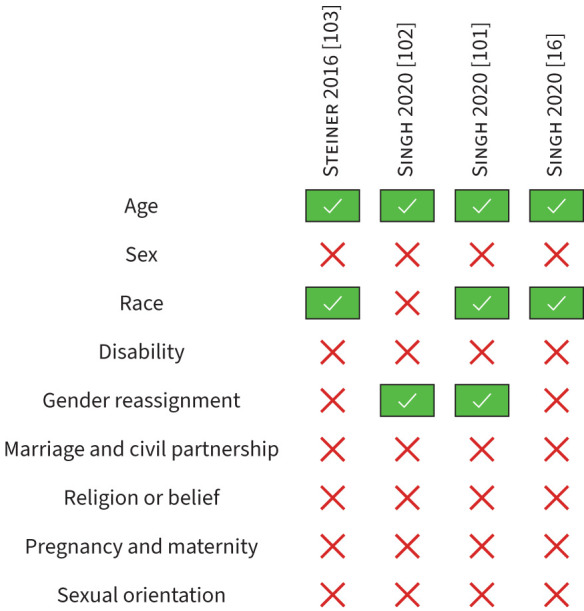
Summary of the protected characteristics reported in the included audits.

Figure 3 summarises the protected characteristics reported by the included pulmonary rehabilitation audits. Further reporting details are detailed in the supplementary material (supplement E).

#### Age

Of the four included audits, 100% reported age (k=4). All audits reported age as median (IQR) (71 (64–76) years [16, 101], 70 (64–76) years [102, 103]), with one audit additionally reporting age as mean±sd (69±9 years) and using frequency data of age categories (<65 years (30%), 65–74 years (42%), 75–84 years (25%) and ≥85 years (4%)) [103].

#### Sex

No audits explicitly reported sex. One (25%) audit reported male and female categories without explicitly stating if these were sex or gender [103] and one (25%) audit reported gender with male and female categories [16].

#### Gender reassignment

Two (50%) audits reported gender with male, female, transgender, other and not recorded/prefers not to say categories [101, 102].

#### Disability

No audits explicitly reported that their service users had a disability or did not have a disability as defined by the UK Equality Act 2010 associated with their COPD. Breathlessness, disease severity, and comorbidities, which may be considered measures of disability, were reported by all audits [16, 101–103]. These included MRC dyspnoea scale (k=4, 100%) as a marker of breathlessness [16, 101–103], FEV_1_ (k=4, 100%) [16, 101–103] and FEV_1_/forced vital capacity (k=3, 75%) [16, 101, 102] as markers of disease severity, and cardiovascular disease (k=4, 100%) [16, 101–103], history of lower limb or lower back musculoskeletal disorders (k=3, 75%) [16, 101, 102] and mental illness (k=3, 75%) [16, 101, 102] as comorbidities.

#### Race

No audits used the term “race”. The ethnicity of service users attending pulmonary rehabilitation was reported in 75% (k=3) of audits [16, 101, 103]. In two (50%) audits, only the White British ethnic group was reported, with this ethnic group being the majority of the service users (94% [103] and 82.5% [16]). One audit reported 16 ethnicity categories (in addition to “not stated”), with the most common categories being White British (82.5%), not stated (11.4%), White Irish (1.5%) and any other White background (1.3%) [101].

#### Marriage and civil partnership, pregnancy and maternity, religion or belief and sexual orientation

No audits reported the characteristics of marriage and civil partnership, pregnancy and maternity, religion or belief and sexual orientation.

## Discussion

This scoping review has identified that protected characteristics are not commonly reported or are inconsistently reported in UK pulmonary rehabilitation research studies and audits. Of the 45 included research studies and audits, age was well collected and reported. Sex was inconsistently reported in research studies. No audits reported sex and usually reported the gender of service users, with half of audits using categories that allowed the expression of gender reassignment. No research studies or audits explicitly reported disability, marriage and civil partnership, pregnancy and maternity, religion or belief or sexual orientation. Without consistently reporting protected characteristics, health inequalities relating to an individual's characteristics in pulmonary rehabilitation will remain unclear.

In order to understand any health inequalities that may exist, appropriate data must be collected [104–106]. In this review of UK research studies and audits, overall audits reported more data on service users’ characteristics than research studies, which may be due to audits reporting on population-level data. However, the audits do not report how much data is missing, so it is unclear how well service users’ characteristics are collected across the UK by pulmonary rehabilitation services inputting data into the audit. No research studies or audits explicitly reported data on disability, marriage and civil partnership, pregnancy and maternity, religion or belief or sexual orientation. By not consistently reporting protected characteristics in pulmonary rehabilitation research studies and audits, the characteristics of individuals accessing and not accessing pulmonary rehabilitation are unclear. As a result, the representativeness, equity and inclusivity of pulmonary rehabilitation across individual characteristics, and therefore health inequalities, are unknown. Furthermore, more evidence is needed to determine whether the intervention is equally effective across all characteristics [107, 108]. To address this, the collection of data is vital [38]. Any results from collecting the data can be used to promote health equity by proposing where evidence-based changes are required in practices, policies and programmes [109], for example in adapting pulmonary rehabilitation to improve uptake and adherence in those least likely to attend [39]. As data on these characteristics have not been commonly collected, then any health inequalities among these characteristics cannot be identified or monitored and improvements cannot be made [104, 109–111]. The significant economic costs of health inequalities cannot be reduced without investing in programmes that target systematically identified health inequalities [33]. Social determinants of health (sex, race/ethnicity, income, education, occupation and social class) [112] are also underreported in advanced chronic respiratory interventions (including pulmonary rehabilitation), meaning that inequalities related to these characteristics that may limit access or completion of respiratory interventions cannot be addressed [111]. Additionally, by capturing data on participants’ gender identity (including if an individual identifies as having the protected characteristic of gender reassignment) and sexual orientation, it empowers individuals (if they wish to disclose this information) with these lived experiences and promotes equality and inclusion [110, 113].

Inconsistency in the reporting of protected characteristics was evident in some pulmonary rehabilitation research studies and audits, particularly when reporting sex. The terms “sex” and “gender” were used interchangeably in several research studies and gender was reported with only male and female categories in approximately a quarter of research studies and audits. No studies or audits explicitly reported nonbinary gender identities or intersex individuals, who account for 0.06% of the UK population [114] and 1.7% of the global population [115], respectively. It is possible that the data reported in male and female genders included other gender identities, and that male and female sex categories included intersex individuals. Sex and gender are different concepts [116–118] and there are more than two gender identities [113, 118]. Sex and gender are commonly used interchangeably and incorrectly in society [118–120] and in health research [121]. However, it is important to understand the difference as there are sex-specific and gender-specific determinants of health [117, 120]. In all research studies and audits that reported race, the term “ethnicity” was used. An individual self-identifies their ethnicity using factors such as culture, language, religion and nationality [122, 123]. In contrast, race is determined by physical characteristics and is a social construct used to group people [123]. Ethnicity is the term used by the UK Census [124], which may explain why ethnicity was reported instead of race in the UK. In other parts of the world, “ethnic identity” (Australia, Bulgaria, Canada, Czech Republic, Hungary, Ireland, Israel, Latvia, Lithuania, New Zealand, Romania, Russia, Slovakia, Slovenia, UK, the United States) and “indigenous identity” (Australia, Canada, Chile, Colombia, Costa Rica, Mexico, New Zealand, the United States) are used to report ethnicity [125].

Where ethnicity was reported, the categories used to report ethnicity varied. In half of audits only the White British ethnic group was reported, but when more than one ethnic group was reported, the number of ethnic groups reported varied considerably. The vast majority of service users in the research studies and audits identified as White British. Ethnic minority groups being underrepresented in research is not exclusive to pulmonary rehabilitation research. Underrepresentation of ethnic minority groups has also been reported in cardiovascular disease [107, 126], diabetes [127] and coronavirus disease 2019 (COVID-19) [128, 129] research. However, if an intervention has been assessed in participants who are predominantly White British, it cannot be assumed that it will be equally effective across all other ethnicities [107, 108]. Furthermore, reduced representation of ethnic minority groups in trials has been found to have wider implications, such as reduced trust and uptake of COVID-19 vaccines in ethnic minority groups [130], which may apply to other interventions or services, such as pulmonary rehabilitation. In a recent scoping review assessing the reporting of race/ethnicity as a social determinant of health in advanced chronic respiratory interventions, ethnicity was also found to be reported in a very small number of studies, limiting the understanding of factors that may be influencing health inequalities [111].

To improve the collection of individual characteristics, such as the nine protected characteristics, reporting frameworks for research studies and audits would be beneficial. Only characteristics that an individual was comfortable to disclose should be collected; however, studies have shown that the majority of service users are willing to provide their characteristics in healthcare settings [131–133]. The method of collecting these data should also be considered (*e.g.* a clinician or researcher asking for the information or an individual completing a form with this information) to ensure that an individual feels comfortable. Data governance should ensure individual anonymity in data reports where characteristics are rare. Reporting frameworks and guidelines have been proposed for sex and gender [134, 135] and ethnicity [136, 137], but no framework or guidelines have been widely adopted in pulmonary rehabilitation. The International Committee of Medical Journal Editors has stated that researchers should strive for participants who are representative and inclusive in age, sex and ethnicity across all study types and provide descriptive data of these characteristics as a minimum [138]. Local frameworks would be required to ensure the population demographics collected meet legislation and the needs of the population. For example, it is illegal to collect religion and ethnicity data in France [139] and the collection of ethnicity data is highly limited in Austria, Belgium, Germany, Luxembourg, Portugal, Slovenia, Sweden and Turkey [125], so a reporting framework for pulmonary rehabilitation in these countries would need to reflect this. Local frameworks could also include additional categories to be collected if relevant to the region or country, in order to meet the needs of the population eligible for pulmonary rehabilitation. This would improve the transparency of participants characteristics in pulmonary rehabilitation and allow the accessibility, representativeness, inclusivity and effectiveness of pulmonary rehabilitation to be assessed appropriately to the local area. This could improve pulmonary rehabilitation delivery by providing information to guide service improvements that can advance equality of opportunity in accessing health services [37] and potentially reduce health inequalities. Further research on any barriers to healthcare professionals and researchers collecting an individuals’ characteristics would be beneficial.

In order to assess the characteristics of who is attending pulmonary rehabilitation, we suggest that all protected characteristics should be collected and made available upon request. However, as a minimum, to report and monitor health inequalities, we would recommend local frameworks report based on the following guidance.

Age: this should be reported as an average with distribution, where possible.

Sex and/or gender: clarity reporting sex and/or gender is essential and terms should be used consistently. If reporting a single sex, the majority sex should be reported. Pay consideration to all gender identity categories, and be clear in reporting if the categories reported represent the entire sample or if smaller categories have been omitted from reporting.

Disability: an individual's perception of their disability should be reported (*e.g*. does their respiratory condition have an impairment on their ability to complete usual day-to-day activities?) and may be collected through measures of the functional ability (*e.g.* MRC score).

Ethnicity: if legislation allows, the ethnicity of individuals should be reported. All ethnic groups, using language and categories that are appropriate to the region or country, should be reported, rather than a single ethnic group. If ethnic groups have been clustered, justifications for this should be provided. In ethnic groups where the number of individuals is small enough that it may identify individuals, the numbers or percentages should not be reported, but this should be explained by the authors.

Other characteristics that are important for the local population: other characteristics that are important to the region or country's health inequalities (socioeconomic status, urban/rural, *etc*.) should be collected. This should be reported in line with national guidance where the research was conducted.

This scoping review assessed individual's characteristics collected and reported in pulmonary rehabilitation research studies and audits using the UK's protected characteristics as an example. Ensuring that pulmonary rehabilitation study participants and service users reflect the diversity of the population eligible for pulmonary rehabilitation and the principle of not discriminating against individuals based on their characteristics is applicable globally. However, other countries may have different policies compared to the UK, and with different health inequalities unrelated to protected characteristics, which need to be accounted for. Furthermore, although protected characteristics are not commonly reported in UK pulmonary rehabilitation studies and audits, this may not reflect a lack of diversity, but a lack of data collected. Future work should include the development of local frameworks for collecting and reporting individual's characteristics and exploring the equity of access, completion and outcomes across such groups in order to investigate health inequalities in pulmonary rehabilitation.

In conclusion, protected characteristics are either not commonly reported or are inconsistently reported in UK pulmonary rehabilitation research studies and audits. Local reporting frameworks for individual's characteristics in pulmonary rehabilitation may support in identifying the health inequalities to be addressed in pulmonary rehabilitation research and services.

**Points for clinical practice**

By collecting and reporting protected characteristics in pulmonary rehabilitation research studies and audits, services may be able to better adapt to meet the needs of those who are not currently accessing services, which may reduce health inequalities.

## Supplementary material

10.1183/16000617.0236-2023.Supp1 **Please note:** supplementary material is not edited by the Editorial Office, and is uploaded as it has been supplied by the author.
Supplementary material ERR-0236-2023.SUPPLEMENT
