# Supplementary material for: Protected characteristics reported in pulmonary rehabilitation: a scoping review
Source: Eur Respir Rev. 2024 Jun 12;33(172):230236. doi: 10.1183/16000617.0236-2023 (PMC11262624; doi:10.1183/16000617.0236-2023)
Supplement: Supplementary file 1 [file ERR-0236-2023.SUPPLEMENT.pdf]

Supplement A: Search strategy

**MEDLINE: (all fields)**
1. “Pulmonary Rehabilitation” OR “Lung Rehabilitation” OR “Respiratory Rehabilitation”
2. Published 2010 or later
3. #1 AND #2
4. Limit #3 to English
5. Limit #4 to All adult
**Scopus: (article title, abstract, keywords)**
(TITLE-ABS-KEY (“Pulmonary Rehabilitation” OR “Lung Rehabilitation” OR “Respiratory Rehabilitation”) AND PUBYEAR >2009 AND (LIMIT-TO (LANGUAGE, "English"))
**CINAHL: (all fields)**
1. “Pulmonary Rehabilitation” OR “Lung Rehabilitation” OR “Respiratory Rehabilitation”
2. Published 2010 or later
3. #1 AND #2
4. Limit #3 to English
5. Limit #4 to All adult
**APA PsycINFO: (all fields)**
1. “Pulmonary Rehabilitation” OR “Lung Rehabilitation” OR “Respiratory Rehabilitation”
2. Published 2010 or later
3. #1 AND #2
4. Limit #3 to English
5. Limit #4 to All adult
**MedRxiv: (title and abstract)**
"Pulmonary Rehabilitation" OR “Lung Rehabilitation” OR “Respiratory Rehabilitation” and posted between "01 Oct, 2010 and 26 Jan, 2024"

Supplement B: Data extraction for the included research studies.

Article Information				Protected Characteristics Reported												
First Author	Year	Evidence Source	Area of Evidence	Age	Age Reporting Details	Sex	Sex Reporting Details	Disability	Disability Reporting Details	Gender Reassignment	Marriage and Civil Partnership	Pregnancy and Maternity	Race	Race Reporting Details	Religion or Belief	Sexual Orientation
Yohannes	2022 a	Published	Clinical	✓	Mean (SD) completers 71.74 (8.84) non-completers 68.22 (10.44). Mean age overall 70.82 (in abstract/results)	?	51% male overall. Non-completers 136 (53% male), 123 (47% female). Referred to as ‘gender’ in table and text and ‘sex’ in the text.	✗	ISWT, FEV_1% predicted, mMRC, DASS depression, DASS anxiety, DASS-stress, FEV_1/FVC, AIR score, SGRQ (symptoms, impact, activity, total),	✗	✗	✗	✗	In discussion: "our COPD population is predominantly Caucasian"	✗	✗
Yohannes	2022 b	Published	Clinical	✓	Mean (SD) total 71.74 (8.84)	?	Gender n (%) male 374 (51) female 360 (49).	✗	FEV_1/FVC, FEV_1% predicted, ISWT, mMRC, SGRQ (symptoms, activity, impact, total)	✗	✗	✗	✗	In discussion: "all patients were Caucasians in this single center trial"	✗	✗
Yohannes	2021	Published	Clinical	✓	Mean (SD) Respondent 72.2 (8.6), Nonrespondent 71.9 (8.4)	✓	Male n (%): Respondent 90 (55), Nonrespondent 20 (57)	✗	AIR score, FEV $_1$% predicted, mMRC, DASS depression, DASS anxiety, DASS stress, SGRQ (symptoms, activity, impact, total), ISWT	✗	✗	✗	✗	In discussion: "predominantly Caucasian patients"	✗	✗

**Article Information**				**Protected Characteristics Reported**													
**First Author**	**Year**	**Evidence Source**	**Area of Evidence**	**Age**	**Age Reporting Details**	**Sex**	**Sex Reporting Details**	**Disability**	**Disability Reporting Details**	**Gender Reassignment**	**Marriage and Civil Partnership**	**Pregnancy and Maternity**	**Race**	**Race Reporting Details**	**Religion or Belief**	**Sexual Orientation**
Wynne	2020	Published	Clinical	✓	Mean (SD) bronchiectasis 70 (10) COPD 70 (9)	?	Gender (female, n (%)) bronchiectasis 38 (61) COPD 46 (58)	✗	MRC, HADS-D, HADS-A, FEV_1, FEV_1/FVC, BMI, FEV_1% predicted, smoking status (current, former, never), mean number of hospital bed days in previous year, number of chest infections in previous year, ISWT, HADS-A, HADS-D, CRQ (D, F, E, M, total).	✗	✗	✗	✗		✗	✗
Ward	2021	Published	Clinical	✓	Mean (SD) 66.3 (7.0)	✓	Male n (%): 11 (58)	✗	FEV_1%predicted, MRC (1-5), BMI, oxygen use (%), smoking status (current, former, never), ISWT, ESWT, QMVC, CRQ (D,F,E,M)	✗	✗	✗	✗		✗	✗
Ricketts	2022	Published	Clinical	✓	Median (IQR) Overall 54 (47–64) year	✓	Overall n (%): female 57 (60) male 38 (40)	✗	Smoking (ex, non-smoker, current), pack-years, atopy, allergic rhinitis, perennial rhinitis, nasal polyps, nasal surgery, eczema, GORD, DB/VCD, psychological illness,	✗	✗	✗	✗		✗	✗

Article Information				Protected Characteristics Reported												
First Author	Year	Evidence Source	Area of Evidence	Age	Age Reporting Details	Sex	Sex Reporting Details	Disability	Disability Reporting Details	Gender Reassignment	Marriage and Civil Partnership	Pregnancy and Maternity	Race	Race Reporting Details	Religion or Belief	Sexual Orientation
									emphysema, bronchiectasis, SAFS/ABPA, DM, HTN, CVD, osteopenia/osteoporosis, SABA nebs, LAMA, ICS/LABA, BDP equivalent dose, prednisolone maintenance and dose, montelukast, theophylline, azithromycin, omalizumab, mepolizumab, antihistamine, nasal steroid, PPI/H2A, MRC, A+E/ICU/hospital admissions in last year, GP attendances in last year, exacerbations in last year, BMI, ACQ6, ACQ overall, AQLQ (symptoms, activity, emotional, environmental), HADS-							

Article Information				Protected Characteristics Reported												
First Author	Year	Evidence Source	Area of Evidence	Age	Age Reporting Details	Sex	Sex Reporting Details	Disability	Disability Reporting Details	Gender Reassignment	Marriage and Civil Partnership	Pregnancy and Maternity	Race	Race Reporting Details	Religion or Belief	Sexual Orientation
									A, HADS-D, eosinophils, PEFR, pre-BD FEV_1% predicted/FEV_1/FVC, 6MWT, BORG score post 6MWT, accelerometery (inactive time, time in LPA, time in MVPA).							
Pavitt	2020	Published	Clinical	✓	Median (IQR) placebo 68 (62,74) Nitrate-rich BRJ 70 (64,78)	?	Gender n (% female): Placebo 26 (41), Nitrate-rich BRJ 27 (42)	✗	Smoking (pack-years), BMI, FEV_1, FEV_1% predicted, GOLD stage (2/3/4), fat free mass, fat free mass index, ISWT, inhalers n (%) LABA, LAMA, LABA-ICS, LABA-LAMA, MRC, CAT score, HADS-A, HADS-D, systolic BP, diastolic BP, MAP, physical activity level, step count per day, time >3METS (min)	✗	✗	✗	✗		✗	✗
Patel	2021	Published	Clinical	✓	Mean (SD) Whole group: PR-min 71 (10), PR-gym 71	✓	Male n (%) Whole group: PR-min 145	✗	FEV_1, FEV_1% predicted, GOLD staging (a/b/c/d), BMI, home	✗	✗	✗	✗		✗	✗

Article Information				Protected Characteristics Reported												
First Author	Year	Evidence Source	Area of Evidence	Age	Age Reporting Details	Sex	Sex Reporting Details	Disability	Disability Reporting Details	Gender Reassignment	Marriage and Civil Partnership	Pregnancy and Maternity	Race	Race Reporting Details	Religion or Belief	Sexual Orientation
					(9)		(45.6), PR gym 157 (49.4)		oxygen user n (%), smoking status (current, former, never), MRC n IQR, ISWT, CRQ (D, F,E,M, total), Charlson comorbidity index, self-reported exacerbation in past year, self-reported hospital days in previous year.							
Patel	2019	Published	Clinical	✓	Mean (SD) Bronchiectasis 70 (11), COPD 71 (10)	✓	n (%) Male Bronchiectasis 77 (36), COPD 79 (37)	✗	FEV_1/FVC, FEV_1% predicted, MRC score, BMI, smoking history in pack-years, exacerbations in past year median IQR, ISWT, CRQ (D, F, E, M, total).	✗	✗	✗	✗		✗	✗
Patel	2023	Published	Clinical	✓	Median (25th, 75th centiles) 72.5 (70, 74.75)	✓	Male n (%) 8 (33%)	✗	BMI, MRC dyspnoea score, FEV_1% predicted, FVC% predicted, resting SpO2, resting heart rate, ISWT (metres,	✗	✗	✗	✗		✗	✗

Article Information				Protected Characteristics Reported													
First Author	Year	Evidence Source	Area of Evidence	Age	Age Reporting Details	Sex	Sex Reporting Details	Disability	Disability Reporting Details	Gender Reassignment	Marriage and Civil Partnership	Pregnancy and Maternity	Race	Race Reporting Details	Religion or Belief	Sexual Orientation
									change SpO2, change heart rate, change BORG), 6MST (change SpO2, change heart rate, change BORG), CPET (VO_2 peak, peak watts, change heart rate, change BORG).							
O'Neill	2018	Publishe d	Clinical	✓	Mean (SD) Whole population 64.4 (8.6) years	?	Gender, male: female 24:25	✗	FEV_1, FEV_1, FEV_1% predicted, FEV_1/FVC, GOLD (mild, moderate, severe, very severe), daily steps, ISWT	✗	✗	✗	✗		✗	✗
Nolan	2022	Publishe d	Clinical	✓	Mean (SD) IPF 73 (9), COPD 73 (8)	✓	n (%) IPF Male 110 (67), COPD 111(68)	✗	BMI, MRC, ISWT, CRQ total, FEV_1/FVC, FEV_1%, FVC%, prescribed supplemental oxygen n (%), ABOT, resting SpO2, smoking history (current, former, never) hospitalised in last year n (%), antibiotics for respiratory tract infection in past year n (%), prescribed	✗	✗	✗	✗		✗	✗

**Article Information**				**Protected Characteristics Reported**												
**First Author**	**Year**	**Evidence Source**	**Area of Evidence**	**Age**	**Age Reporting Details**	**Sex**	**Sex Reporting Details**	**Disability**	**Disability Reporting Details**	**Gender Reassignment**	**Marriage and Civil Partnership**	**Pregnancy and Maternity**	**Race**	**Race Reporting Details**	**Religion or Belief**	**Sexual Orientation**
									antifibrotic therapy, cardiovascular disease, pulmonary hypertension, diabetes n (%), frail, CRQ (D, F, E, M).							
Nolan	2017	Published	Clinical	✓	Mean (SD) Whole group 68 (9)	✓	n (%) male 110 (72%)	✗	FEV_1% predicted, FEV_1/FVC, MRC, smoking status (never, former, current, pack year history), ADO index, COTE index, SpO2 on RA, current medication (LTOT, long/short acting bronchodilators, inhaled corticosteroids, oral steroids (maintenance), ABOT, NIV), BMI, walking aid (%) (none, walking frame or walking stick), 4MGS, ISWT, CRQ (D, F, E, M, total), accelerometer	✗	✗	✗	✗		✗	✗

Article Information				Protected Characteristics Reported												
First Author	Year	Evidence Source	Area of Evidence	Age	Age Reporting Details	Sex	Sex Reporting Details	Disability	Disability Reporting Details	Gender Reassignment	Marriage and Civil Partnership	Pregnancy and Maternity	Race	Race Reporting Details	Religion or Belief	Sexual Orientation
									(moderate intensity PA, daily step count - pedometer and accelerometer).							
Nolan	2019	Published	Clinical	✓	Mean (SD) PR 71 (9), Home 71 (10)	?	Gender (%): male PR 47, home 49.	✗	FEV_1, FEV_1% predicted, MRC, FEV_1/FVC, LTOT (%), ABOT (%), BMI, smoking status (current, former, never), 4MGS, ISW distance, CRQ (D, F, E, M, total).	✗	✗	✗	✗		✗	✗
Nikoletou	2023	Published	Clinical	✓	Mean (SD) total group: 71.0 (11.05), control group 69.8 (10.9), intervention group 70.5 (10.9)	?	Gender (male) (number (%)) total group: 32 (55.2), control group: 14 (56), intervention group: 18 (55)	✗	ILD-GAP index (0-1, 2-3, 4-5, >5), BMI, waist/hip ratio, fat (%), FVC (% predicted), diffusion capacity of lung for carbon dioxide(% predicted), comorbidities- (cardiac, hypertension, diabetes, high cholesterol), prednisolone medication (<5mg, 5-10mg, >10mg),	✗	✗	✗	✗		✗	✗

Article Information				Protected Characteristics Reported													
First Author	Year	Evidence Source	Area of Evidence	Age	Age Reporting Details	Sex	Sex Reporting Details	Disability	Disability Reporting Details	Gender Reassignment	Marriage and Civil Partnership	Pregnancy and Maternity	Race	Race Reporting Details	Religion or Belief	Sexual Orientation
									smoking status (current, former, never, pack year history), 6MWD, SpO2 in room air (%), sniff nasal pressure, PE max, handgrip (dominant hand), quads extension (dominant side), hip flexion (dominant side), HADS (anxiety, depression), SGRQ for IPF							
McDonnell	2014	Published	Clinical	✓	Mean (SD) Observation group completed 68.1 (11.3), controlled group 73.1 (8.5), observation group drop out/excluded 62.67 (11.1).	?	Gender (n, %): Observation group completed male 13 (46), control group 11 (46), observation group drop out/excluded 15 (45).	✗	FEV_1% predicted, FEV_1/FVC, PSQI, CAT, HADS-A, HADS-D, ISWT.	✗	✗	✗	✗		✗	✗

Article Information				Protected Characteristics Reported													
First Author	Year	Evidence Source	Area of Evidence	Age	Age Reporting Details	Sex	Sex Reporting Details	Disability	Disability Reporting Details	Gender Reassignment	Marriage and Civil Partnership	Pregnancy and Maternity	Race	Race Reporting Details	Religion or Belief	Sexual Orientation
Majd	2020	Published	Clinical	✓	Mean (SD) Completers 58 (11), non-completers 50 (15)	✓	n (%) female completers 18 (60) non completers 20 (65)	✗	BMI, MRC median IQR, FEV_1, FEV_1% predicted, FEV_1/FVC, atopic, ICS, oral prednisolone, ACQ, CRQ (D, F, E, F, M), total AQLQ, AQLQ (S, AL, EF, ES, A), HADS-A, HADS-D, ITM VO2pk, ICE VO2pk, peak oxygen uptake. ISWT, QMVC, FeNO, sputum eosinophil, DEXA scan.	✗	✗	✗	✗		✗	✗
Maddocks	2016	Published	Clinical	✓	Mean (SD) All: age 69.8 (9.7) years,	✓	Male n (%) 484 (59.3)	✗	Smoking status (current, former, never), MRC mean (SD), FEV_1% predicted, GOLD stage, HADS-A, HADS-D, self-reported hospital admissions in previous year n %, self-reported number of exacerbations in previous year n%, age adjusted Charlson index, ADO score, BMI,	✗	✗	✗	✗		✗	✗

Article Information				Protected Characteristics Reported												
First Author	Year	Evidence Source	Area of Evidence	Age	Age Reporting Details	Sex	Sex Reporting Details	Disability	Disability Reporting Details	Gender Reassignment	Marriage and Civil Partnership	Pregnancy and Maternity	Race	Race Reporting Details	Religion or Belief	Sexual Orientation
									SMI, sarcopenia, handgrip, peak QMVC, below QMVC cut off point, 4MGS, ISWT, CRQ (D,F,E,M), self-reported weekly energy expenditure, self-reported time in moderate activity, CAT, Katz, frailty characteristic (% meeting criteria)(unintentional weight loss, exhaustion, low physical activity, slow gait speed, weak handgrip strength).							
Lewis	2021	Published	Clinical	✓	Mean (SD) Started online PR 69.7 (10.7), declined online PR 72.9 (10.8)	?	Gender: started online PR male/female 9/8, declined online PR 6/7	✗	MRC, BMI, respiratory diagnosis (COPD/ILD/asthma), 1 min sit to stand, GAD, PHQ9, CRQ (D, F, E, M).	✗	✗	✗	✗		✗	✗
Knox	2019	Published	Clinical	✓	Mean (SD) hub 68.6 (12.8),	?	Hub Female 58.3%, spoke	✗	MRC, HADS-D, HADS-A, respiratory	✗	✗	✗	✗		✗	✗

Article Information				Protected Characteristics Reported													
First Author	Year	Evidence Source	Area of Evidence	Age	Age Reporting Details	Sex	Sex Reporting Details	Disability	Disability Reporting Details	Gender Reassignment	Marriage and Civil Partnership	Pregnancy and Maternity	Race	Race Reporting Details	Religion or Belief	Sexual Orientation
					spoke 70.1 (10.8)		33.3%		diagnosis, CAT, ISWT							
Jung	2020	Published	Clinical	✓	Age of each participant (10 participants) (75, 68, 69, 73, 62,71,76,69,63,63). Mean (SD) male 70 (4.8) female 67 (5.2)	?	Gender of each participant (male and female only) (6 male, 4 female)	✗	MRC 4 or 5, SPPB, CRQ (D, F, E, M), patient activation measure, Edmonton frail scale, PHQ-9, GAD-7.	✗	✗	✗	✗		✗	✗
Jones	2014	Published	Clinical	✗	N/A	✗	N/A	✗	AECOPD over a 12-month period, length of hospital stay median (IQR).	✗	✗	✗	✗		✗	✗
Jones	2015	Published	Clinical	✓	Mean (SD) No sarcopenia 66 (10), low SMI 69 (7), low function only 73 (9), sarcopenia 73 (8)	?	Sex male: female no sarcopenia 147:73, low SMI only 14:13, low function only 136:149, sarcopenia 57:33. 'Gender' in Table 2.	✗	FEV_1% predicted, use of walking aid n%, MRC, LTOT n%, BMI, SMM, SMI, handgrip, peak QMVC, 4MGS, 5STS, SPPB, ISWT, CAT, SGRQ, smoking status (current, former, never), Charlson index, hospital inpatient days in previous 12 months, number of exacerbations in	✗	✗	✗	✗		✗	✗

Article Information				Protected Characteristics Reported												
First Author	Year	Evidence Source	Area of Evidence	Age	Age Reporting Details	Sex	Sex Reporting Details	Disability	Disability Reporting Details	Gender Reassignment	Marriage and Civil Partnership	Pregnancy and Maternity	Race	Race Reporting Details	Religion or Belief	Sexual Orientation
									previous 12 months n IQR, self-report physical activity, objective physical activity.							
Jenkins	2020 a	Published	Clinical	✓	Mean (SD) frequent 69.6 (7) infrequent 69.3 (7.3)	?	Male (%): frequent 58%, infrequent 57%	✗	Body mass, GOLD (grade A/B/C/D), FEV_1% predicted, Charlson comorbidity index, mMRC (0/1/2/3/4), HADS, ISWT, ESWT, CRQ.	✗	✗	✗	✗		✗	✗
Jenkins	2020 b	Published	Clinical	✓	Mean (SD) COPD phase 1 69 (7)	?	Male (%): COPD phase 1 58%	✗	Body mass, GOLD grade (A/B/C/D), FEV_1% predicted, Charlson comorbidity index, current smokers, oxygen users (%), hospitalisation in past 12 months mean (SD), exacerbations in past 12 months mean (SD), daily beclomethasone equivalent mean (SD), mMRC, average	✗	✗	✗	✗		✗	✗

Article Information				Protected Characteristics Reported												
First Author	Year	Evidence Source	Area of Evidence	Age	Age Reporting Details	Sex	Sex Reporting Details	Disability	Disability Reporting Details	Gender Reassignment	Marriage and Civil Partnership	Pregnancy and Maternity	Race	Race Reporting Details	Religion or Belief	Sexual Orientation
									exercise duration in minutes mean (SD).							
France	2021	Published	Clinical	✓	N Median AECOPD (IQR) 68.0 (61.5-74.0), PR 69 (64.0-73.0)	✓	Male n (%) AECOPD 27 (60%), PR 37 (55.2%) (described as sex in 3.1)	✗	FEV_1 median IQR, FEV_1% predicted median IQR, FEV_1/FVC, MRC n % (2-5B), Smoking status (never, ex, current), pack years, O2 use (n ,%), hospital admission in last 12 months (n, %), exacerbations in last 12 months (n,%), co-morbidities (n,%) (cardiac disease, hypertension, diabetes, kidney disease, arthritis/ musculoskeletal, mental health disorder, malignancy, HADS-D, HADS-A, CAT, SPPB, CRQ (D, E, M, F).	✗	✗	✗	✗		✗	✗
Finnegan	2023	Published	Clinical	✓	Median (range) d-cycloserine 71.0 (47-81),	✓	91 participants (30 female).	✗	Smoking pack years, total exacerbations, BMI, MRC	✗	✗	✗	✗		✗	✗

Article Information				Protected Characteristics Reported												
**First Author**	**Year**	**Evidence Source**	**Area of Evidence**	**Age**	**Age Reporting Details**	**Sex**	**Sex Reporting Details**	**Disability**	**Disability Reporting Details**	**Gender Reassignment**	**Marriage and Civil Partnership**	**Pregnancy and Maternity**	**Race**	**Race Reporting Details**	**Religion or Belief**	**Sexual Orientation**
					placebo 71.5 (46-85)		Later referred to as sex in the text.		breathlessness scale, resting SpO2%, resting heart rate, FEV_1/FVC, FEV_1% predicted, GOLD (1(a/b/c/d), 2 (a/b/c/d), 3 (a/b/c/d), 4 (a/b/c/d), comorbidities (asthma, hypertension, gastro-oesophageal reflux, swelling of both ankles, surgery to the chest, depression, diabetes, heart attack, bronchiectasis, osteoporosis, arrhythmia, inflammatory bowel disease, peptic ulcer, heart failure, tuberculosis).							
Edwards	2023	Published	Clinical	✓	Mean (SD) 73 (9)	?	Male n (%) 163 (69)	✗	FVC, FVC (%predicted), FEV_1/FVC, MRC, BMI, ischemic heart disease, pulmonary hypertension,	✗	✗	✗	✗		✗	✗

Article Information				Protected Characteristics Reported												
**First Author**	**Year**	**Evidence Source**	**Area of Evidence**	**Age**	**Age Reporting Details**	**Sex**	**Sex Reporting Details**	**Disability**	**Disability Reporting Details**	**Gender Reassignment**	**Marriage and Civil Partnership**	**Pregnancy and Maternity**	**Race**	**Race Reporting Details**	**Religion or Belief**	**Sexual Orientation**
									prescribed antifibrotic therapy, prescribed medication for anxiety, prescribed medication for depression, LTOT, ambulatory oxygen therapy, HADS (anxiety, depression), ISWT, CRQ							
Cox	2018	Publishe d	Economi c	✓	Mean, (median, SD, range). Across all trial arms: 67, 68,9,40-85) Page 89. Table 7 (Pg36) mean (SD) overall 67.8 (11.12)	✓	Table 7 (Pg39) Male n (%) 22 (39%), female 35 (61). Pg 89 Sex female 25 (57%).	✗	FEV_1, FEV_1% predicted, co-morbidities (ischaemic, stroke, vascular, diabetes) (n, %), Extended MRC, London Chest Activity of Daily Living Scale, EQ5D, CAT, Dyspnoea, Eosinopenia, Consolidation, Acidemia and AF score, Perceived Necessity and Concerns Questionnaire, Malnutrition Universal Screening Tool, 6MWD, health and	✗	✗	✗	✗		✗	✗

Article Information				Protected Characteristics Reported													
First Author	Year	Evidence Source	Area of Evidence	Age	Age Reporting Details	Sex	Sex Reporting Details	Disability	Disability Reporting Details	Gender Reassignment	Marriage and Civil Partnership	Pregnancy and Maternity	Race	Race Reporting Details	Religion or Belief	Sexual Orientation
									social care resource use questionnaire.							
Chaplin	2017	Published	Clinical	✓	Mean (SD) PR 66.1 (8.1), WEB 66.4 (10.1)	?	Gender (%): PR male 63.5, WEB 74.5	✗	FEV_1% predicted, BMI, MRC (IQR and n) 2-5, ISWT, ESWT, CRQ SR-D, CAT, PRAISE, HADS-A, HADS-D, BCKQ, EQ5D.	✗	✗	✗	✗		✗	✗
Chaplin	2015	Published	Clinical	✓	Mean (SD) stable 71.1 (8.9), post exacerbation 70.6 (8.6)	✓	n (%) stable: male 74 (59%) female 51 (41%), Post exacerbation: male 23 (31%), female 52 (69%).	✗	BMI, MRC median (IQR), FEV_1, CRQ (D, F, M, E), HADS-A, HADS-D, CAT, ISWT, ESWT.	✗	✗	✗	✗		✗	✗
Chaplin	2022	Published	Clinical	✓	Mean (SD) web-based PR 68.3 (6.5), conventional PR 67.4 (8.6)	?	Gender (male /female) (n): web-based PR 18/2, conventional 19/15.	✗	FEV_1, FEV_1% predicted, home oxygen use (n, %), MRC, BMI, smoking status (current, non-smoker, ex-smoker, unknown), ISWT, ESWT.	✗	✗	✗	✗		✗	✗
Chalmers	2019	Published	Clinical	✓	Patients enrolled at baseline Median (IQR) 68	✓	Female n (%) 31 (64.6%)	✗	Co-morbidities (angina, MI, osteoporosis, anxiety,	✗	✗	✗	✗		✗	✗

Article Information				Protected Characteristics Reported												
First Author	Year	Evidence Source	Area of Evidence	Age	Age Reporting Details	Sex	Sex Reporting Details	Disability	Disability Reporting Details	Gender Reassignment	Marriage and Civil Partnership	Pregnancy and Maternity	Race	Race Reporting Details	Religion or Belief	Sexual Orientation
					(63-72)				diabetes, HTN), smoking status (ever, ex, current), Medications (inhaled corticosteroids, macrolide, other long-term antibiotics, inhaled antibiotics), exacerbations per year (1,2,3 or more), BMI, FEV_1, FEV_1% predicted, FVC, bronchiectasis severity index (mild, moderate, severe), 6-minute walk distance, SGRQ, Leicester cough questionnaire, CAT.							
Briggs-Price	2022	Published	Clinical	✓	Mean (SD) PR comparison 68.2 (10.54) n=1394 Hip and knee pain 69.3 (8.91) n=97	?	Male (n, %) PR comparison 762, 52. Hip and knee pain 54, 55.7.	✗	FEV_1%, BMI, MRC grade (1-5), primary respiratory diagnosis (n, %), ISWT, ESWT, MRC, HADS-A, HADS-D.	✗	✗	✗	✗		✗	✗
Bradley	2022	Published	Clinical	✓	Mean age of 72	✓	Six female and three male	✗	No measures reported	✗	✗	✗	✗		✗	✗

Article Information				Protected Characteristics Reported													
First Author	Year	Evidence Source	Area of Evidence	Age	Age Reporting Details	Sex	Sex Reporting Details	Disability	Disability Reporting Details	Gender Reassignment	Marriage and Civil Partnership	Pregnancy and Maternity	Race	Race Reporting Details	Religion or Belief	Sexual Orientation
Boutou	2014	Published	Clinical	✓	Mean (SD) 68.1 (10.5)	✓	Total population 49.6% male.	✗	GOLD staging, FEV_1% predicted, FEV_1/FVC, MRC, HAD-A, HAD-D, BMI, ISWT, 6MWT, CAT, CRDQ (D, E, F, M).	✗	✗	✗	✗		✗	✗
Bourne	2017	Published	Clinical	✓	Mean (SD) F2F 71.4 (8.6), online PR 69.1 (7.9)	?	Gender n (%): face-to-face male 18 (69), online PR 41 (62).	✗	COPD severity (n, %) (mild, moderate, severe, very severe), FEV_1, FEV_1% predicted, FVC, FVC predicted, co-morbidities (n, %) (hypertension, CVD, dermatological, diabetes and endocrine, gastroenterological, haematological, neurological and psychiatric, history of malignancy, MSK, renal, other respiratory, none), 6MWT, CAT, HADS, SGRQ, mMRC.	✗	✗	✗	✗		✗	✗
Barlow	2020	Published	Clinical	✓	Mean (SD) Total 71.7 (8.1) range	?	Gender (n): 134 male, 99	✗	COPD severity (mild, moderate, severe, very	✗	✗	✗	✗		✗	✗

Article Information				Protected Characteristics Reported												
First Author	Year	Evidence Source	Area of Evidence	Age	Age Reporting Details	Sex	Sex Reporting Details	Disability	Disability Reporting Details	Gender Reassignment	Marriage and Civil Partnership	Pregnancy and Maternity	Race	Race Reporting Details	Religion or Belief	Sexual Orientation
					45-90		female.		severe) n, CAT, CCQ, ISWT, HADS-A, HADS-D.							
Armstrong	2021	Published	Clinical	✓	Mean (SD) PR alone: 73 (9), PR and physical activity: 71 (9)	?	Gender (male/female) PR alone: 9/15, PR and physical activity: 9/15	✗	BMI, FEV_1, FEV_1 (% predicted), FEV_1/FVC, steps per day, 6MWD, mMRC, HADS (anxiety and depression)	✗	✗	✗	✗		✗	✗
Andrews	2015	Published	Clinical	✓	Mean (SD) 6-week PR 69 (10), 7-week PR 72 (9), 8-week PR 72 (10)	?	Gender n (%): overall total population female 163 (48%), male 178 (52%)	✗	COPD severity (mild, moderate, severe, very severe, other) (n, %, smoking status (current smoker, ex-smoker, never smoked).	✗	✗	✗	✓	Ethnicity n (%): 6-week PR: White British 106 (75), Irish 0(0), White other 1 (1), Mixed British 15 (11), African 2 (1). 7-week PR: White British 61 (77), Irish 1(1), White other 1 (1), Mixed British 0 (0), African	✗	✗

Article Information				Protected Characteristics Reported												
First Author	Year	Evidence Source	Area of Evidence	Age	Age Reporting Details	Sex	Sex Reporting Details	Disability	Disability Reporting Details	Gender Reassignment	Marriage and Civil Partnership	Pregnancy and Maternity	Race	Race Reporting Details	Religion or Belief	Sexual Orientation
														0 (0).<br>8-week PR: White British 127 (89), Irish 1(1), White other 0 (0), Mixed British 1 (1), African 0 (0).		
Aldabayan	2019	Published	Clinical	✓	Mean (SD) total population 71.31 (9.06)	✓	Total population n (%): male 59 (58%), female 43 (42%).	✗	Smoking status (active/ex), Smoking history (pack years), BMI, FEV_1, FEV_1% predicted, FEV_1/FVC%, aortic pulse wave velocity, systolic BP, diastolic BP, MAP, pulse pressure, ISWT, mMRC, CAT, HADS-A, HADS-D, CV risk determinants (diabetes, hypertension, hyperlipidaemia, IHD, MI, peripheral arterial disease, HF, AF,	✗	✗	✗	✗		✗	✗

Article Information				Protected Characteristics Reported													
First Author	Year	Evidence Source	Area of Evidence	Age	Age Reporting Details	Sex	Sex Reporting Details	Disability	Disability Reporting Details	Gender Reassignment	Marriage and Civil Partnership	Pregnancy and Maternity	Race	Race Reporting Details	Religion or Belief	Sexual Orientation
									stroke).							

Abbreviations: BRJ: beetroot juice, ISWT: incremental shuttle walking test, FEV_1: forced expiratory volume in one second, mMRC: modified Medical Research Council, DASS: Depression Anxiety Stress Scales , FEV_1/FVC: forced expiratory volume in one second/forced vital capacity, AIR: Anxiety Inventory for Respiratory Disease, SGRQ: St George's Respiratory Questionnaire, COPD: chronic obstructive pulmonary disease, MRC: medical research council, HADS-D: Hospital Anxiety and Depression Scale - Depression, HADS-A: Hospital Anxiety and Depression Scale – Anxiety, BMI: body mass index, CRQ (D, F, E, M): Chronic Respiratory Disease Questionnaire (dyspnoea, fatigue, emotional function, mastery), FEV_1% predicted: forced expiratory volume in one second as a percentage of the predicted value, ESWT: endurance shuttle walking test, QMVC: quadriceps maximal voluntary contraction , GORD: gastro-oesophageal reflux disease, DB/VCD: dysfunctional breathing/vocal cord dysfunction, SAFS: severe asthma with fungal sensitisation, ABPA allergic bronchopulmonary aspergillosis, DM: diabetes mellitus, HTN: hypertension, CVD: cardiovascular disease, SABA: short-acting beta agonist, LAMA: long-acting muscarinic antagonists , ICS: inhaled corticosteroids , LABA: long-acting beta agonists, BDP: beclometasone diproprionate dose equivalent, PPI: proton pump inhibitors, H2A: histamine-2 receptor antagonists, A+E: accident and emergency, ICU: intensive care unit, GP: general practitioner , ACQ: Asthma Control Questionnaire , AQLQ: Asthma Quality of Life Questionnaire , PEFR: peak expiratory flow rate , pre-BD: pre-bronchodilator, 6MWT: 6-minute walk test, LPA: light intensity physical activity, MVPA: moderate to vigorous physical activity, GOLD: Global Initiative for Obstructive Lung Disease, CAT: Chronic Obstructive Pulmonary Disease Assessment Test, BP: blood pressure, MAP: mean arterial pressure, METS: metabolic equivalents , ABOT: ambulatory oxygen therapy, SpO2: saturation of peripheral oxygen, ADO index: age, dyspnoea, airflow obstruction index, COTE index: Chronic Obstructive Pulmonary Disease specific co-morbidity index , LTOT: long-term oxygen therapy , NIV: non-invasive ventilation, 4MGS: 4-metre gait speed, PA: physical activity, ISW: incremental shuttle walk, PSQI: Pittsburgh Sleep Quality Index , ITM: Incremental exercise test on a treadmill, VO2pk: peak oxygen uptake, ICE: Incremental exercise test on a cycle ergometer , FeNO: fractional exhaled nitric oxide , DEXA: dual-energy x-ray absorptiometry, SMI: skeletal muscle mass index , GAD: generalised anxiety disorder , PHQ9: Patient Health Questionnaire-9, SPPB: Short Physical Performance Battery , GAD-7: Generalized Anxiety Disorder-7, AECOPD: acute exacerbation of chronic obstructive pulmonary disease, SMM: skeletal muscle mass, 5STS: 5 times sit to stand test, EQ5D: Euro-Qol – 5 dimension, 6MWD: 6-minute walking distance, CRQ SR-D: Chronic Respiratory Disease Questionnaire Self-Reported Dyspnoea, PRAISE: Pulmonary Rehabilitation Adapted Index of Self Efficacy, BCKQ: Bristol Chronic Obsturctive Pulmonary Disease Knowledge Questionnaire , MI: myocardial infarction, CRDQ: Chronic Respiratory Disease Questionnaire , MSK: musculoskeletal, CCQ: Clinical Chronic Obstructive Pulmonary Disease Questionnaire, CV: cardiovascular, IHD: ischemic heart disease, HF: heart failure, AF: atrial fibrillation, PR: pulmonary rehabilitation, ILD-GAP: interstitial lung disease – gender, age, physiology, PE max: maximal expiratory pressure, CPET: cardiopulmonary exercise test.

Supplement C: Data extraction for the included National Respiratory Audit Programme (NRAP) / National Asthma and Chronic Obstructive Pulmonary Disease Audit Programme (NACAP) articles.

NRAP Audit Information		Protected Characteristics Reported														
First Author	Year	Age	Age Reporting Details	Sex	Sex Reporting Details	Disability	Disability Reporting Details	Gender Reassignment	Gender Reassignment Reporting Details	Marriage and Civil Partnership	Pregnancy and Maternity	Race	Race Reporting Details	Religion or Belief	Sexual Orientation
Singh	2020a	✓	Median age at referral 71 (IQR (64-76))	✗	Gender n (%): Male 3128 (51.7%), female 2922 (48.2%), transgender 1 (0%), other 2 (0%), not recorded/prefer not to say 3 (0%).	✗	MRC, FEV1/FVC, FEV1, CAT, CRQ, cardiovascular disease, history of lower limb or lower back MSK disorders, mental illness (anxiety, depression, severe mental illness), BMI, smoking status.	✓	Gender n (%): Male 3128 (51.7%), female 2922 (48.2%), transgender 1 (0%), other 2 (0%), not recorded/ prefer not to say 3 (0%).	✗	✗	✓	Ethnicity n (%): African 16 (0.1%), any other Asian background 32 (0.3%), any other black background 21 (0.2%), any other ethnic group 39 (0.3%), any other mixed background 35 (0.3%), any other white background 153 (1.3%), Bangladeshi 24 (0.2%), Caribbean 56 (0.5%), Chinese 6 (0.0%), Indian 84 (0.7%), not stated 1382 (11.4%),	✗	✗

NRAP Audit Information		Protected Characteristics Reported													
First Author	Year	Age	Age Reporting Details	Sex	Sex Reporting Details	Disability	Disability Reporting Details	Gender Reassignment	Gender Reassignment Reporting Details	Marriage and Civil Partnership	Pregnancy and Maternity	Race	Race Reporting Details	Religion or Belief	Sexual Orientation
													Pakistani 48 (0.4%), White and Asian 9 (0.1%), White and black African 12 (0.1%), White and Black Caribbean 23 (0.2%), White British 10,005 (82.5%), White Irish 182 (1.5%).		
Singh	2020b	✓	Median age at referral 71 (IQR (64-76))	?	Males 52.8% and females 47.1%	✗	MRC, FEV_1/FVC, FEV_1, CAT, CRQ, cardiovascular disease, history of lower limb or lower back MSK disorders, mental illness, smoking status,	✗	N/A	✗	✗	✓	82.5% White British	✗	✗
Singh	2019	✓	Median age at referral 70 (IQR (64-76))	✗	Gender n (%): Male 3128 (51.7%), female 2922 (48.2%), transgender 1 (0%), other 2 (0%), not recorded/prefer not to say 3 (0%).	✗	MRC, FEV1/FVC, FEV1, CAT, CRQ, cardiovascular disease, history of lower limb or lower back MSK disorders, mental	✓	Gender n (%): Male 3128 (51.7%), female 2922 (48.2%), transgender 1 (0%), other 2 (0%), not	✗	✗	✗		✗	✗

NRAP Audit Information		Protected Characteristics Reported													
First Author	Year	Age	Age Reporting Details	Sex	Sex Reporting Details	Disability	Disability Reporting Details	Gender Reassignment	Gender Reassignment Reporting Details	Marriage and Civil Partnership	Pregnancy and Maternity	Race	Race Reporting Details	Religion or Belief	Sexual Orientation
							illness (anxiety, depression, severe mental illness), BMI, smoking status.		recorded/ prefer not to say 3 (0%).						
Steiner	2016	✓	Mean (SD) 69 (9), Median (IQR) 70 (64-76), 30% under 65, 42% 65-74, 25% 75-84, 4% 85 or older	?	Gender: 53% males and 47% females	✗	MRC, FEV1, FEV1% predicted, BMI, GOLD Stage, SGRQ, CAT, CRQ, haemoglobin saturation at rest, oxygen usage at home, significant medical conditions (see table 2.2) e.g., diabetes, gastrointestinal condition, kidney disease etc., number of times hospitalised with AECOPD in past 12 months, NIV at home, smoking status.	✗	N/A	✗	✗	✓	"Ethnicity was known for 94% of the audit sample. When known, 94% were recorded as being White British."	✗	✗

IQR: Interquartile Range, FEV1/FVC: forced expiratory volume in one second/forced vital capacity, FEV1: forced expiratory volume in one second, MSK: musculoskeletal, MRC: Medical Research Council, BMI: body mass index, SGRQ: St George's Respiratory Questionnaire, CAT: COPD Assessment Test, CRQ: Chronic Respiratory Disease Questionnaire, GOLD: Global Initiative for Obstructive Lung Disease, AECOPD: acute exacerbation of Chronic Obstructive Pulmonary Disease, NIV: non-invasive ventilation.

Supplement D: The protected characteristics and reporting details in the included research studies

	Protected Characteristic												
	Age		Sex		Race		Disability	Gender Reassignment	Marriage and Civil Partnership	Sexual Orientation	Religion or Belief	Pregnancy and Maternity
First Author and Year	Reported (✓/ ✗)	Reporting Details	Reported (✓/✗/ ?)*	Reporting Details	Reported (✓/✗)	Reporting Details	Reported (✓/✗)	Reported (✓/✗)	Reported (✓/✗)	Reported (✓/✗)	Reported (✓/✗)	Reported (✓/✗)
Aldabayan 2019	✓	Mean (SD)	✓	Male and female (n, %)	✗	N/A	✗	✗	✗	✗	✗	✗
Andrews 2015	✓	Mean (SD)	?	Gender: male and female (n, %)	✓	Ethnicity: White British, Irish, White other, Mixed British and African (n, %)	✗	✗	✗	✗	✗	✗
Armstrong, 2021	✓	Mean (SD)	?	Gender: male/female (n)	✗	N/A	✗	✗	✗	✗	✗	✗

	Protected Characteristic													
	Age		Sex		Race		Disability	Gender Reassignment	Marriage and Civil Partnership	Sexual Orientation	Religion or Belief	Pregnancy and Maternity
**First Author and Year**	**Reported (✓/X)**	**Reporting Details**	**Reported (✓/X/?)***	**Reporting Details**	**Reported (✓/X)**	**Reporting Details**	**Reported (✓/X)**	**Reported (✓/X)**	**Reported (✓/X)**	**Reported (✓/X)**	**Reported (✓/X)**	**Reported (✓/X)**
Barlow 2020	✓	Mean (SD)	?	Gender: male and female (n)	X	N/A	X	X	X	X	X	X
Bourne 2017	✓	Mean (SD)	X	Gender: male (n, %)	X	N/A	X	X	X	X	X	X
Boutou 2014	✓	Mean (SD)	✓	Male and female (n, %)	X	N/A	X	X	X	X	X	X
Bradley, 2022	✓	Mean	✓	Female and male (n)	X	N/A	X	X	X	X	X	X
Briggs-Price 2022	✓	Mean (SD)	?	'Sex' and 'gender' used interchangeably. Male (n, %)	X	N/A	X	X	X	X	X	X

	Protected Characteristic											
	Age		Sex		Race		Disability	Gender Reassignment	Marriage and Civil Partnership	Sexual Orientation	Religion or Belief	Pregnancy and Maternity
**First Author and Year**	**Reported (✓/ Ⅹ)**	**Reporting Details**	**Reported (✓/Ⅹ/ ?)***	**Reporting Details**	**Reported (✓/Ⅹ)**	**Reporting Details**	**Reported (✓/Ⅹ)**	**Reported (✓/Ⅹ)**	**Reported (✓/Ⅹ)**	**Reported (✓/Ⅹ)**	**Reported (✓/Ⅹ)**	**Reported (✓/Ⅹ)**
Chalmers 2019	✓	Median (IRQ)	✓	Female (n, %)	Ⅹ	N/A	Ⅹ	Ⅹ	Ⅹ	Ⅹ	Ⅹ	Ⅹ
Chaplin 2022	✓	Mean (SD)	?	Gender: male and female (n)	Ⅹ	N/A	Ⅹ	Ⅹ	Ⅹ	Ⅹ	Ⅹ	Ⅹ
Chaplin 2015	✓	Mean (SD)	✓	Male and female (n, %)	Ⅹ	N/A	Ⅹ	Ⅹ	Ⅹ	Ⅹ	Ⅹ	Ⅹ
Chaplin 2017	✓	Mean (SD)	Ⅹ	Gender: male (%)	Ⅹ	N/A	Ⅹ	Ⅹ	Ⅹ	Ⅹ	Ⅹ	Ⅹ
Cox 2018	✓	Mean (SD), range and median	✓	Male and female (n, %)	Ⅹ	N/A	Ⅹ	Ⅹ	Ⅹ	Ⅹ	Ⅹ	Ⅹ
Edwards, 2023	✓	Mean (SD)	?	Male (n, %)	Ⅹ	N/A	Ⅹ	Ⅹ	Ⅹ	Ⅹ	Ⅹ	Ⅹ

	Protected Characteristic											
	Age		Sex		Race		Disability	Gender Reassignment	Marriage and Civil Partnership	Sexual Orientation	Religion or Belief	Pregnancy and Maternity
**First Author and Year**	**Reported (✓/✗)**	**Reporting Details**	**Reported (✓/✗/?)***	**Reporting Details**	**Reported (✓/✗)**	**Reporting Details**	**Reported (✓/✗)**	**Reported (✓/✗)**	**Reported (✓/✗)**	**Reported (✓/✗)**	**Reported (✓/✗)**	**Reported (✓/✗)**
Finnegan, 2023	✓	Median (range)	✓	Female (n). Referred to as sex in text	✗	N/A	✗	✗	✗	✗	✗	✗
France 2021	✓	Median (IRQ)	✓	Male (n, %)	✗	N/A	✗	✗	✗	✗	✗	✗
Jenkins 2020a	✓	Mean (SD)	?	Male (%)	✗	N/A	✗	✗	✗	✗	✗	✗
Jenkins 2020b	✓	Mean (SD)	?	Male (%)	✗	N/A	✗	✗	✗	✗	✗	✗
Jones 2015	✓	Mean (SD)	?	‘Sex’ and ‘gender’ used interchangeably. Male and female	✗	N/A	✗	✗	✗	✗	✗	✗

	**Protected Characteristic**											
	**Age**		**Sex**		**Race**		**Disability**	**Gender Reassignment**	**Marriage and Civil Partnership**	**Sexual Orientation**	**Religion or Belief**	**Pregnancy and Maternity**
**First Author and Year**	**Reported (✓/✗)**	**Reporting Details**	**Reported (✓/✗/?)***	**Reporting Details**	**Reported (✓/✗)**	**Reporting Details**	**Reported (✓/✗)**	**Reported (✓/✗)**	**Reported (✓/✗)**	**Reported (✓/✗)**	**Reported (✓/✗)**	**Reported (✓/✗)**
				(n)								
Jones 2014	✗	N/A	✗	Not reported	✗	N/A	✗	✗	✗	✗	✗	✗
Jung 2020	✓	Age of each participant reported	?	Reported if each participant had a male or female gender.	✗	N/A	✗	✗	✗	✗	✗	✗
Knox 2019	✓	Mean (SD)	?	Female (%)	✗	N/A	✗	✗	✗	✗	✗	✗
Lewis 2021	✓	Mean (SD)	?	Gender: male and female (n)	✗	N/A	✗	✗	✗	✗	✗	✗
Maddocks 2016	✓	Mean (SD)	✓	Male (n, %)	✗	N/A	✗	✗	✗	✗	✗	✗

	Protected Characteristic											
	Age		Sex		Race		Disability	Gender Reassignment	Marriage and Civil Partnership	Sexual Orientation	Religion or Belief	Pregnancy and Maternity
First Author and Year	Reported (✓/✗)	Reporting Details	Reported (✓/✗/?)*	Reporting Details	Reported (✓/✗)	Reporting Details	Reported (✓/✗)	Reported (✓/✗)	Reported (✓/✗)	Reported (✓/✗)	Reported (✓/✗)	Reported (✓/✗)
Majd 2020	✓	Mean (SD)	✓	Female (n, %)	✗	N/A	✗	✗	✗	✗	✗	✗
McDonnell 2014	✓	Mean (SD)	✗	Gender: male (n, %)	✗	N/A	✗	✗	✗	✗	✗	✗
Nikoletou, 2023	✓	Mean (SD)	✗	Gender: male (n, %)	✗	N/A	✗	✗	✗	✗	✗	✗
Nolan 2019	✓	Mean (SD)	✗	Gender: male (%)	✗	N/A	✗	✗	✗	✗	✗	✗
Nolan 2017	✓	Mean (SD)	✓	Male (n, %)	✗	N/A	✗	✗	✗	✗	✗	✗
Nolan 2022	✓	Mean (SD)	✓	Male (n, %)	✗	N/A	✗	✗	✗	✗	✗	✗
O’Neill 2018	✓	Mean (SD)	?	Gender: male and female (n)	✗	N/A	✗	✗	✗	✗	✗	✗
Patel 2023	✓	Median	✓	Male (n, %)	✗	N/A	✗	✗	✗	✗	✗	✗

	Protected Characteristic														
	Age		Sex		Race		Disability	Gender Reassignment	Marriage and Civil Partnership	Sexual Orientation	Religion or Belief	Pregnancy and Maternity
**First Author and Year**	**Reported (✓/✗)**	**Reporting Details**	**Reported (✓/✗/?)***	**Reporting Details**	**Reported (✓/✗)**	**Reporting Details**	**Reported (✓/✗)**	**Reported (✓/✗)**	**Reported (✓/✗)**	**Reported (✓/✗)**	**Reported (✓/✗)**	**Reported (✓/✗)**
		(25th, 75th centiles)										
Patel 2021	✓	Mean (SD)	✓	Male (n, %)	✗	N/A	✗	✗	✗	✗	✗	✗
Patel 2019	✓	Mean (SD)	✓	Male (n, %)	✗	N/A	✗	✗	✗	✗	✗	✗
Pavitt 2020	✓	Median (IRQ)	✗	Gender: female (n, %)	✗	N/A	✗	✗	✗	✗	✗	✗
Ricketts 2022	✓	Median (IRQ)	✓	Male and female (n, %)	✗	N/A	✗	✗	✗	✗	✗	✗
Ward 2021	✓	Mean (SD)	✓	Male (n, %)	✗	N/A	✗	✗	✗	✗	✗	✗
Wynne 2020	✓	Mean	✗	Gender:	✗	N/A	✗	✗	✗	✗	✗	✗

	Protected Characteristic												
	Age		Sex		Race		Disability	Gender Reassignment	Marriage and Civil Partnership	Sexual Orientation	Religion or Belief	Pregnancy and Maternity
**First Author and Year**	**Reported (✓/✗)**	**Reporting Details**	**Reported (✓/✗/?)***	**Reporting Details**	**Reported (✓/✗)**	**Reporting Details**	**Reported (✓/✗)**	**Reported (✓/✗)**	**Reported (✓/✗)**	**Reported (✓/✗)**	**Reported (✓/✗)**	**Reported (✓/✗)**
		(SD)		female (n, %)								
Yohannes 2021	✓	Mean (SD)	✓	Male (n, %)	✗	N/A	✗	✗	✗	✗	✗	✗
Yohannes 2022a	✓	Mean (SD)	?	'Sex' and 'gender' used interchangeably. Male and female (n, %).	✗	N/A	✗	✗	✗	✗	✗	✗

*Table 1: Protected characteristics reported in pulmonary rehabilitation research studies. *For the protected characteristic of sex: ✓: Sex is explicitly reported in the research study. ✗: Sex is not explicitly reported in the research study. ?: It is unclear if the study has reported the protected characteristic of sex (e.g. gender is reported with only sex categories or 'sex' and 'gender' are used interchangeably for the same data or sex or gender categories are described in the research study, but it is not specified what this category is describing).*

Supplement E: The protected characteristics and reporting details in the included audits.

	Protected Characteristic													
	Age		Sex		Race		Disability	Gender Reassignment		Marriage and Civil Partnership	Sexual Orientation	Religion or Belief	Pregnancy and Maternity
First Author and Year	Reported (✓/ᵡ)	Reporting Details	Reported (✓/ᵡ/?)*	Reporting Details	Reported (✓/ᵡ)	Reporting Details	Reported (✓/ᵡ)	Reported (✓/ᵡ)	Reporting details	Reported (✓/ᵡ)	Reported (✓/ᵡ)	Reported (✓/ᵡ)	Reported (✓/ᵡ)
Steiner, 2016	✓	Mean (SD), median (IRQ) and age categories (under 65, 65-74, 75-84 and 85 or older)	?	Gender: male and female (%)	✓	Ethnicity: White British (%)	ᵡ	ᵡ	Not reported	ᵡ	ᵡ	ᵡ	ᵡ
Singh, 2019	✓	Median (IRQ)	ᵡ	Gender: male, female, transgender,	ᵡ	N/A	ᵡ	✓	Gender: male, female, transgender,	ᵡ	ᵡ	ᵡ	ᵡ

	Protected Characteristic													
	Age		Sex		Race		Disability	Gender Reassignment		Marriage and Civil Partnership	Sexual Orientation	Religion or Belief	Pregnancy and Maternity
First Author and Year	Reported (✓/✗)	Reporting Details	Reported (✓/✗/?)*	Reporting Details	Reported (✓/✗)	Reporting Details	Reported (✓/✗)	Reported (✓/✗)	Reporting details	Reported (✓/✗)	Reported (✓/✗)	Reported (✓/✗)	Reported (✓/✗)
				other and prefer not to say/not recorded (%)					other and prefer not to say/not recorded (%)				
Singh, 2020a	✓	Median (IRQ)	✗	Gender: male, female, transgender, other and prefer not to say/not recorded (%)	✓	Ethnicity: African, any other Asian background, any other black background, any other ethnic group, any other mixed	✗	✓	Gender: male, female, transgender, other and prefer not to say/not recorded (%)	✗	✗	✗	✗

	Protected Characteristic												
	Age		Sex		Race		Disability	Gender Reassignment		Marriage and Civil Partnership	Sexual Orientation	Religion or Belief	Pregnancy and Maternity
First Author and Year	Reported (✓/✗)	Reporting Details	Reported (✓/✗/?)*	Reporting Details	Reported (✓/✗)	Reporting Details	Reported (✓/✗)	Reported (✓/✗)	Reporting details	Reported (✓/✗)	Reported (✓/✗)	Reported (✓/✗)	Reported (✓/✗)
						background, any other white background, Bangladeshi, Caribbean, Chinese, Indian, not stated, Pakistani, White and Asian, White and black African, White and							

	Protected Characteristic													
	Age		Sex		Race		Disability	Gender Reassignment		Marriage and Civil Partnership	Sexual Orientation	Religion or Belief	Pregnancy and Maternity
First Author and Year	Reported (✓/✗)	Reporting Details	Reported (✓/✗/?)*	Reporting Details	Reported (✓/✗)	Reporting Details	Reported (✓/✗)	Reported (✓/✗)	Reporting details	Reported (✓/✗)	Reported (✓/✗)	Reported (✓/✗)	Reported (✓/✗)
						black Caribbean, White British, White Irish (n, %)							
Singh, 2020b	✓	Median (IRQ)	?	Male and female (%)	✓	Ethnicity: White British (%)	✗	✗	Not reported	✗	✗	✗	✗

*Table 2: Protected characteristics reported in pulmonary rehabilitation audits. *For the protected characteristic of sex: ✓: Sex is explicitly reported in the research study. ✗: Sex is not explicitly reported in the research study. ?: It is unclear if the protected characteristic of sex is reported (e.g. gender is reported with only sex categories or sex or gender categories are described in the research study, but it is not specified what this category is describing).*
